# An Insight into the Characteristics of 3D Printed Polymer Materials for Orthoses Applications: Experimental Study

**DOI:** 10.3390/polym16030403

**Published:** 2024-01-31

**Authors:** Syed Hammad Mian, Emad Abouel Nasr, Khaja Moiduddin, Mustafa Saleh, Hisham Alkhalefah

**Affiliations:** 1 Advanced Manufacturing Institute, King Saud University, Riyadh 11421, Saudi Arabia; 2 Department of Industrial Engineering, College of Engineering, King Saud University, Riyadh 11421, Saudi Arabia

**Keywords:** orthosis, 3D printing, fused deposition modeling, tensile test, water absorption, microscopic imaging, dimensional accuracy

## Abstract

Knee orthoses assist patients with impaired gait through the amendment of knee abnormalities, restoration of mobility, alleviation of pain, shielding, and immobilization. The inevitable issues with laborious traditional plaster molding procedures for orthoses can be resolved with 3D printing. However, a number of challenges have limited the adoption of 3D printing, the most significant of which is the proper material selection for orthoses. This is so because the material used to make an orthosis affects its strength, adaptability, longevity, weight, moisture response, etc. This study intends to examine the mechanical, physical, and dimensional characteristics of three-dimensional (3D) printing materials (PLA, ABS, PETG, TPU, and PP). The aim of this investigation is to gain knowledge about these materials’ potential for usage as knee orthosis materials. Tensile testing, Olympus microscope imaging, water absorption studies, and coordinate measuring machine-based dimension analysis are used to characterize the various 3D printing materials. Based on the investigation, PLA outperforms all other materials in terms of yield strength (25.98 MPa), tensile strength (30.89 MPa), and shrinkage (0.46%). PP is the least water absorbent (0.15%) and most flexible (407.99%); however, it is the most difficult to fabricate using 3D printing. When producing knee orthoses with 3D printing, PLA can be used for the orthosis frame and other structural elements, PLA or ABS for moving parts like hinges, PP for padding, and TPU or PP for the straps. This study provides useful information for scientists and medical professionals who are intrigued about various polymer materials for 3D printing and their effective utilization to fabricate knee orthoses.

## 1. Introduction

Aging-related functional degradation in the lower limbs, limitations, and gait problems are common in senior persons [1]. This draws a lot of social and ethical attention to the subject of helping the elderly to live more independently and societally, particularly in areas like mobility. An orthosis refers to an externally worn device that has been designed to provide support or correction for muscles that have seen a decrease in strength in various parts of the body, including the spine, arms, knee, feet, and legs [2]. The use of a knee orthosis has been seen to yield notable enhancements in walking characteristics among individuals afflicted with mild-to-moderate osteoarthritis of the medial compartment of the knee [3].

Traditionally, a labor-intensive plaster molding process is used to create knee orthoses [4]. From taking the knee impression to plaster casting, it is a laborious and lengthy procedure that requires specialized equipment such as a casting room, a sink with plaster capture, and a dry space. Positive plaster casts are also bulky and must be handled carefully to avoid breaking and needing to recast the patient. It is a costly technique since, after every cast, the positive cast is destroyed to make room for fresh castings in storage. Furthermore, it is worth noting that the production time for a manually fabricated orthosis is around one week, but a three-dimensional (3D) printer is capable of completing the same work within a single day [5]. These conventional techniques may result in higher expenses and longer patient wait times as they are labor-intensive, limit design options, demand a high degree of expertise, and need specialized infrastructure.

Digital methodologies are increasingly being recognized as viable alternatives to traditional plaster-based fabrication techniques. These approaches have the ability to effectively mitigate issues such as extended production timelines, excessive material use, and high expenses [6]. The process of digital manufacturing known as 3D printing necessitates the inclusion of knee morphology data, which may be acquired by direct 3D scanning of the knee [7]. The digitized data have the potential to be used in the creation of a 3D representation of the patient. Computer-aided design (CAD) software is used for the manipulation of 3D scan data, allowing for modifications to be made to the geometric properties. The 3D model that is obtained from the adjusted positive model or the final orthotic design can be sent to a 3D printer to fabricate the patient-specific knee orthosis.

The inception of 3D printing can be traced back to 1984 when Charles Hull, working at 3D Systems, pioneered the development of stereolithography (SLA) as a manufacturing process [8]. There are many 3D printing processes, including fused deposition modeling (FDM), photopolymer printing, selective laser sintering (SLS), selective laser melting (SLM), and multi-jet modeling (MJM) [9]. The fundamental concept that underlies all additive technologies is uniform: a virtual representation of the intended product is partitioned into a sequence of distinct two-dimensional (2D) layers. These layers are then transformed into a set of sequential instructions that guide a printer in accurately depositing material, one layer at a time, onto a construction platform. The dissimilarities among these procedures lie in the variations in the used materials and the intricacies of the production procedure. The layer-by-layer deposition of polymer filaments, provided by the FDM or fused filament fabrication (FFF) technology, is recognized as a highly accessible and extensively used technological approach [10]. The operational basis of FDM 3D printers involves the extrusion of thermoplastic filaments via a heated nozzle. This process entails the melting of a material and its successive application, resulting in the creation of the final product as per a predetermined model.

Currently, 3D printing is widely utilized across numerous applications and proves to be beneficial in a diverse range of fields including the healthcare sector [11]. The market size of the global 3D printing healthcare industry was valued at USD 2239.73 million in 2023 and is projected to reach USD 5994.38 million by 2030, exhibiting a compound annual growth rate (CAGR) of 17.83% during the forecast period of 2023–2030 [12]. Orthotics and prosthetics constitute a significant portion of these medical devices and have recently started providing cost-effective and highly customized solutions [13]. Despite the considerable potential of additive manufacturing (AM) in the field of manufacturing, there is still a lack of clear legal frameworks that specifically define the status of 3D printed objects, whether they are intended for implantation or for non-implantable use [14]. Another noteworthy constraint regarding the utilization of 3D printing for practical purposes pertains to the accessibility of materials that are appropriate for the particular applications.

Polymer materials, such as polylactic acid (PLA) and polyethylene terephthalate glycol (PETG), are often acknowledged as the thermoplastic materials most frequently used and economically viable in the FDM method, owing to their simplicity and ease of usage [15]. Polyacrylonitrile-butadiene-styrene (ABS), polypropylene (PP), polyamide (PA), polycarbonate (PC), and thermoplastic polyurethane (TPU) are some of the polymeric materials that are also readily available in the marketplace. However, it is crucial to recognize that these materials also possess significant limitations. This implies that these polymers have both advantages and limitations. For example, it is observed that materials such as PA and PC necessitate elevated extrusion temperatures [16]. Likewise, TPU demonstrates a relatively low mechanical modulus [17]. Additionally, it is known that ABS and PC generate toxic substances when subjected to the melting process [18]. Moreover, both PA and PC possess a high density, while PA and ABS encounter notable levels of shrinkage [19]. Moreover, it is worth noting that both PA and PC contain significant quantities of hazardous fillers and additives [20]. It is also important to acknowledge that PA, PC, and TPU are linked to higher costs [19].

The effectiveness and performance of a knee orthosis are significantly influenced by factors such as the manufacturing process, design components, and materials used. The use of high-quality materials and advanced manufacturing procedures has the capacity to decrease replacement costs and mitigate amputee injuries, while concurrently improving the fit and functioning. The physical characteristics of a knee orthotic device, such as elasticity, resilience, flexibility, durability, hardness, density, and temperature responsiveness, are ultimately determined by the kind of material used [21]. The mechanical requirements of the orthosis are dictated by its intended medical use, whereby considerations such as load-bearing capacity and shear stress resistance are pivotal aspects. In the field of orthotics, it is essential for implant materials to possess the ability to endure repeated loading and unloading cycles subjected to diverse stresses, including bending, twisting, and shearing stress. Furthermore, orthotic devices are subjected to prolonged exposure to corrosive conditions, which may have an adverse effect on their qualities. Hence, it is vital to conduct a precise assessment of the mechanical characteristics of these materials in order to mitigate fractures and uphold good functionality [22]. This assessment offers significant insights into the material’s capacity to endure and adjust to external stresses, hence informing the design and selection of the orthosis that can effectively fulfill the mechanical demands of their intended uses. 

Commonly used materials for the fabrication of 3D printed knee orthoses include PLA, ABS, TPU, PETG, PP, etc. PLA is a substance known for its ecologically sustainable characteristics since it is devoid of any environmental hormones or heavy metals. Moreover, it is worth noting that PLA has remarkable attributes in terms of its renewability and biocompatibility, as shown by previous research [23]. ABS constitute a styrene resin variant, characterized by its composition of these three constituent elements. ABS has notable characteristics such as exceptional impact resistance and convenient processability [24]. The PETG material is often favored as a biomaterial for medical devices due to its superior stability range, enabling printing at lower temperatures and/or quicker rates [19]. TPU has notable mechanical qualities, including the most elevated tensile strength, tearing strength, and abrasion resistance when compared to other thermoplastic elastomers [25]. PP finds widespread use across several domains within the medical industry. It is a low-cost polymer that is perfect for a variety of applications due to its outstanding qualities, which include flame resistance, transparency, high thermal distortion temperature, dimensional stability, and recyclability [26]. Consequently, these materials are extensively used in the production of orthoses by exceptional endurance. These materials have been employed in the FDM process for the fabrication of orthoses by multiple researchers [27,28,29]. To choose the right material for a given application, it is essential to consider the magnitude of mechanical and environmental loads that the application would entail, as well as the geometric limitations associated with the object’s printing process.

However, it is important to note that the choice of materials for orthotics should not rely just on one mechanical characteristic of the product. When a material is selected based on its mechanical performance, there is a considerable probability that the expected physical attributes, biomechanical effectiveness, or the desired level of comfort may not be attained. The selection of materials and the final design of the product are dependent on the chosen fabrication technique. Hence, it is important to recognize the specific materials that are appropriate for the process of AM. The selection of materials is a result of a trade-off between the range of materials that are accessible and the constraints imposed by the prevailing printing method. In order to achieve the intended outcome, it is important to thoroughly assess parameters such as mechanical strength, water absorption extent, dimensional accuracy, etc. Undoubtedly, the selection of materials is a critical stage in the development of a novel medical device such as an orthosis.

The present work focuses on a comprehensive characterization of 3D printed samples composed of five distinct materials for the application of knee orthosis. This characterization encompasses an examination of the thermal, mechanical, physical, and dimensional characteristics of the 3D printing materials (PLA, ABS, PETG, TPU, and PP). The aim of this investigation is to gain knowledge about these materials’ potential for usage as knee orthosis materials.

This work is novel since it conducts a thorough experimental investigation of five distinct 3D printing polymer materials. To find a material that can meet the needs of an orthosis, several materials have been examined and their performance has been evaluated. This study intends to fill a gap in the literature by examining different rigid and flexible polymer materials for orthosis use. This work is particularly significant to the research community involved in orthosis design and 3D printing for the following reasons. This research explores the relevant performance metrics that need to be considered when choosing a material for 3D printing an orthosis. It lays out a methodology for the effective and efficient experimental measurement of performance parameters, such as tensile strength, yield strength ratio, shrinkage, water absorption, etc. A thorough analysis of the fracture behaviors of the polymer materials is conducted, and the results can be very helpful for developing a reliable orthosis. This study makes contributions to the domains of engineering and medicine by outlining the benefits and drawbacks of various 3D printing polymers and their suitability for orthotic applications. It aids in identifying the materials useful for an orthosis, how to quantify those materials experimentally in an efficient manner, and understanding how these materials may fail. 

## 2. Materials and Methods

**Figure 1:**
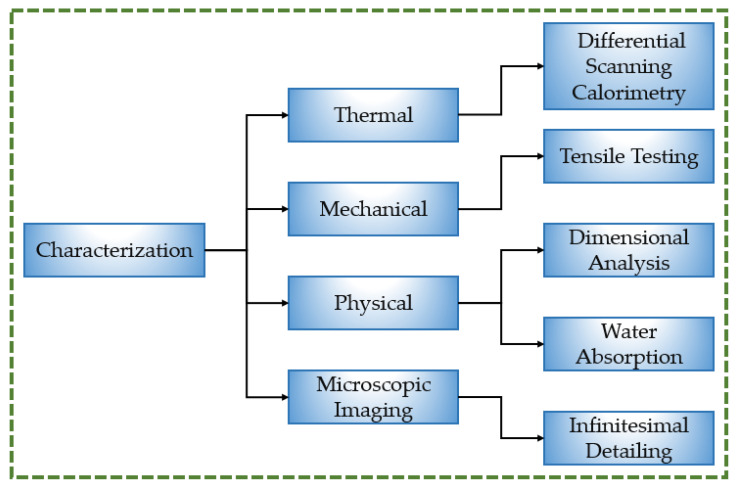
Methodology adopted to characterize various orthosis materials.

The present study is distinctive in that it examines five different 3D printing materials: PLA, ABS, PETG, TPU, and PP. The goal was to determine their suitability for the production of knee orthoses. The methodology to characterize the different materials is depicted in Figure 1. The initial testing procedure was thermal characterization using differential scanning calorimetry (DSC). It was employed to ascertain the melting and glass transition temperatures of the materials examined in this study. The materials were also subjected to tensile testing because tensile strength determines an orthosis’s effectiveness to withstand an externally applied load without falling apart. This implies that a material’s appropriateness increases with its tensile strength. To understand the fracture mechanics and the cause of a certain type of fracture in a given material, tensile testing was followed by microscopic imaging. One of the prerequisites for a material’s durability in orthotics is its ability to withstand water. A device’s ability to withstand or repel the negative effects of water and prevent its absorption explain its moisture resistance. This study therefore analyzed water absorption for various materials. A dimensional analysis was also conducted to ascertain the dimensional variations in the materials upon their 3D printing. One of the biggest problems with components manufactured using 3D printing is dimensional inaccuracy. A more accurate fit, higher comfort, and improved ease when performing daily duties are all benefits of a highly accurate orthosis.

**Figure 2:**
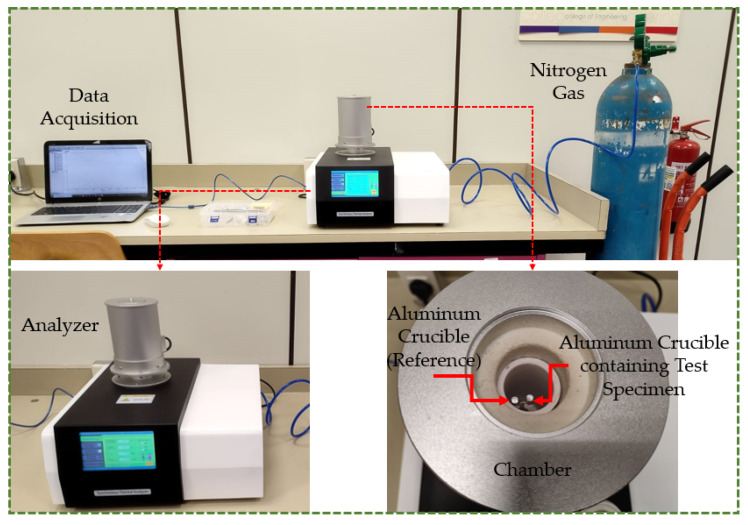
Differential scanning calorimetry setup.

DSC testing was undertaken to characterize the glass transition (T_g_) and melting temperatures (T_m_) of the test materials. PLA, ABS, PETG, TPU, and PP filaments as acquired were utilized for this testing, and samples weighing around 10 mg were used. The test was carried out using an LR-STA200 Synchronous Thermal Analyzer (Lonory, Dongguan, China) in compliance with ASTM D3418 [30,31,32]. As depicted in Figure 2, the test specimens were placed in aluminum crucibles, and the temperature was firstly adjusted to ambient temperature (around 20–25 °C). The temperature was then scaled from the ambient temperature to an end temperature at a rate of 10 °C/min. The end temperature depended on the material being tested. For instance, it was 220 °C for PLA, and 250 °C for ABS, PETG, TPU, and PP. The end test temperatures were controlled according to supplier information and the literature. Nitrogen gas was supplied to the chamber at a flow rate of 50 mL/min to prevent any contamination during the test. The acquired DSC curve was employed to determine the glass transition and melting temperatures.

**Figure 3:**
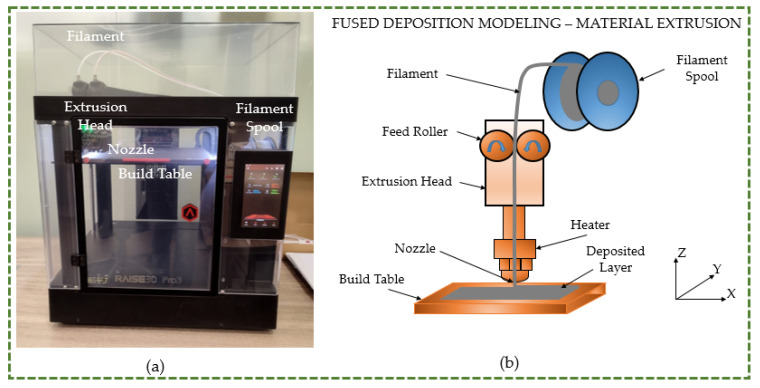
(**a**) 3D printer machine. (**b**) Schematic representation of fused deposition modeling.

The test specimens were built using an open-filament 3D printer known as Raise 3D pro 3 (Raise 3D Technologies Inc., Irvine, CA, USA), as illustrated in Figure 3a. Figure 3b depicts the fused deposition modeling (FDM) process schematics, demonstrating the operating principle of Raise 3D. The extrusion head, which was driven by an electric motor, was attached to the filaments held in the spool. The build platform travelled in the Z-direction, while the extrusion head travelled in the X- and Y-directions. Generally, the filament diameter ranges from 1.75 to 3.0 mm [33]. The three steps of the FDM fabrication technique are pre-processing, manufacturing, and post-processing. During the pre-processing phase, the specimen’s design was produced by utilizing computer-aided design (CAD) software and saved in standard tessellation language (STL) format.

The STL file was imported into the IdeaMaker software program (v4.3.3, Raise 3D Technologies Inc., Irvine, CA, USA) to generate the G-code instructions for the 3D printer. All of the necessary process parameters, including the heated bed temperature, nozzle temperature, layer height, speed, infill percentage, etc., were set before slicing the file and generating the G-code. The settings for these variables in the current research were chosen in accordance with the company’s specifications. The reason for this is that the filaments were also purchased from Raise 3D, which conducted extensive testing to establish the profiles for each material. The default temperature and speed settings were chosen for each material since they are the most effective for printing the materials. Table 1 displays the parameters for fabricating different materials.

**Figure 4:**
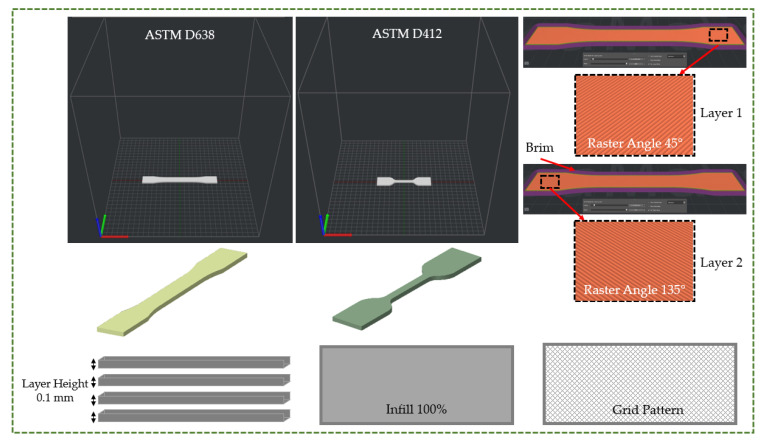
Illustration of the printing parameters [34,35].

The test specimens were built flatwise along the X-direction, as seen in Figure 4. The raster angle of the grid infill pattern was changed by 90° between two successive layers. The infill grid pattern was created using the raster angles 45° and 135°. The infill percentage and layer height were maintained at 100% and 0.1 mm, respectively. Five specimens of each material were produced and tested to guarantee the results’ repeatability and remove any bias. The measuring results of five samples were averaged to determine the final assessment.

Following the pre-processing phase, temperature control was used to heat the incoming material from the spool to the head until it attained a semi-liquid state. Over the build platform, two-dimensional (2D) layers were formed, and these layers were placed upon one another to produce a 3D specimen. As indicated in Table 1, the build platform and filaments were heated in accordance with the designated bed and nozzle temperatures for each material. After each layer was printed, the building platform descended, the extrusion process was continued, and the final product was created. The 3D printed border known as the “Brim”, which protruded from the edges of the 3D printed object, was removed from the final product in the post-processing phase. The purpose of the brim was indeed to provide the 3D printed object with a greater surface area so that it adhered to the print bed appropriately.

**Figure 5:**
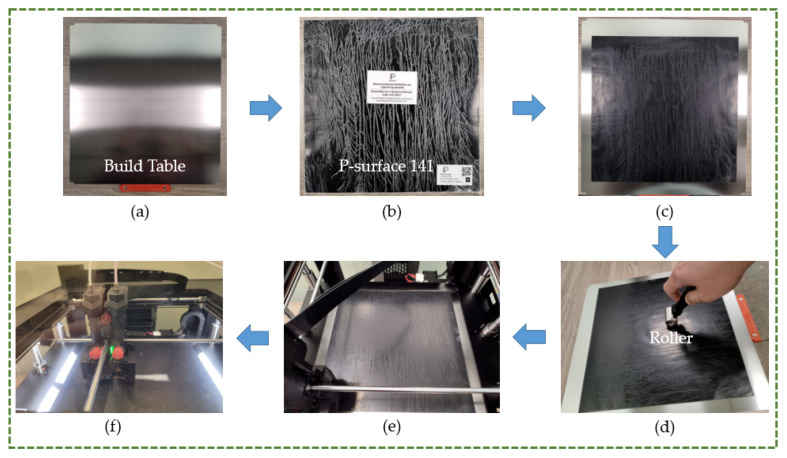
(**a**) Build table. (**b**) P-surface 141. (**c**) P-surface 141 placed on build table. (**d**) Installation using roller. (**e**) PP platform inside machine. (**f**) PP part 3D printing.

TPU is generally harder to print than PLA, ABS, and PETG because of its increased flexibility. It must be printed at a slower print speed than other materials to prevent stretching and tangling. Higher speeds may result in under-extrusion or compression of the filament, which may clog, jam, or string. Similarly, PP is challenging to print because of its greater flexibility and poorer adherence to the build table. Henceforth, as Table 1 illustrates, PP was built at a slower speed than the rigid materials to increase its printability. Additionally, as illustrated in Figure 5, a unique surface called P-surface 141 (PPprint GmbH, Bayreuth, Germany) was used for PP to improve adherence to the build table. The installation of P-surface on the build platform is shown in Figure 5a–f. After the P-surface was positioned on the building platform, it had to be properly attached there using a roller and light pressure, as seen in Figure 5d. Additionally, in order to remove the finished product without damaging it, it was necessary to heat the printer bed to 110 °C. 

**Figure 6:**
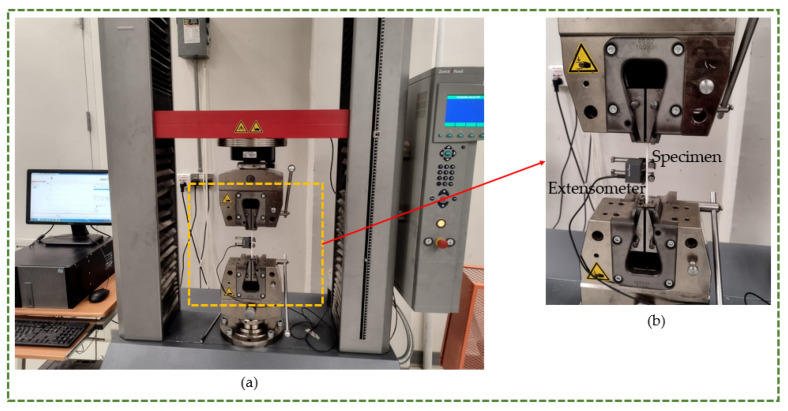
(**a**) Universal tensile testing machine. (**b**) Enlarged view of the setup.

The mechanical properties were characterized by tensile testing. Tensile testing was performed on the printed specimens in accordance with ASTM guidelines. The tensile standards for rigid and flexible filaments are ASTM D638 [34] and ASTM D412 [35], respectively [36]. In the current research, the flexible filaments were TPU and PP, while the remaining filaments were rigid. For PLA, ABS, and PETG, the ASTM D638 standard specimen was utilized, whereas the ASTM D412 standard specimen was adopted for TPU and PP. As shown in Figure 6a, a Zwick Z100 electromechanical universal testing machine (ZwickRoell, Ulm, Germany) equipped with a 100 kN load cell was used to measure tensile strength, yield strength, and elongation at break. An extensometer was utilized to measure the modulus of elasticity experimentally within the elastic range (see Figure 6b). The extensometer was secured to the tensile test specimen to measure the elongation, which assisted in the precise estimation of Young’s modulus [37]. The yield point, which marks the transition from the material’s elastic to plastic behavior, was challenging to obtain. It implies that the material becomes permanently plastically distorted if the yield threshold is exceeded. The 0.2% strain offset approach is a useful technique that is mostly used to determine yield strength [38]. Accordingly, the stress value that corresponds to the 0.2% plastic strain is the yield strength (0.2% offset). Proof stress is another term for this. Consequently, this work’s yield strength was calculated utilizing the 0.2% offset approach [39]. Testing of the rigid specimens, comprising PLA, ABS, and PETG, was conducted at a speed of 5 mm/min according to the D638 Type I standard [34,40,41]. The TPU and PP specimens were evaluated in accordance with ASTM D412 using a combination of 5 mm/min and 200 mm/min speeds [42,43]. A speed of 5 mm/min was maintained up until the yield point, at which point it was increased to 200 mm/min.

**Figure 7:**
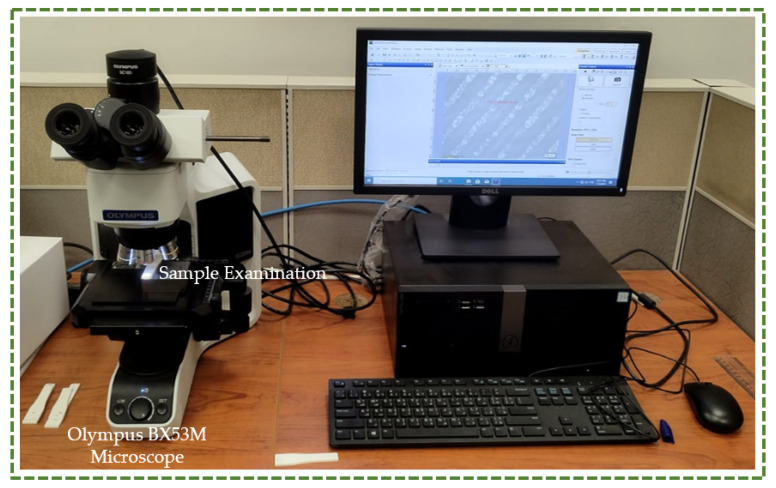
Optical microscope setup for infinitesimal detailing.

The test specimens were also examined using an optical microscope to characterize the surface of the fabricated specimens and comprehend the causes of a certain type of fracture. An optical microscope Olympus BX53M (Olympus, Tokyo, Japan) was utilized for this purpose as well as for microscopic observation [44,45]. As illustrated in Figure 7, an upright optical microscope was able to capture optical images with a ×100 lens, illuminating the components from beneath [46,47]. In this study, optical pictures were taken at a 5× magnification because the field of view was more limited than the region being examined.

**Figure 8:**
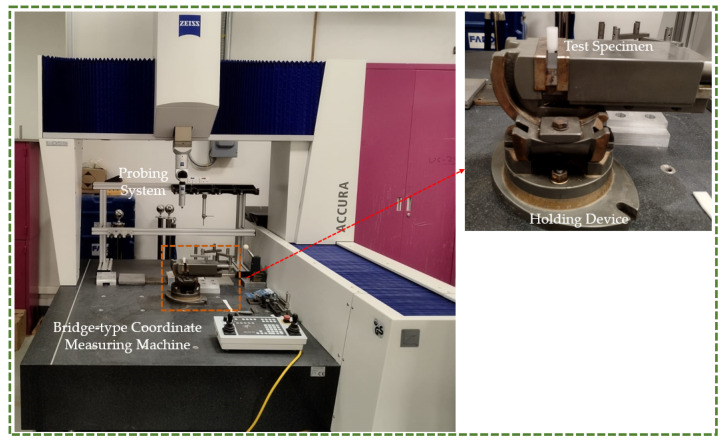
Dimension analysis using CMM.

A bridge-type Coordinate Measuring Machine (CMM) with a touch-trigger probe (ACCURA, Zeiss, Oberkochen, Germany) was deployed, as shown in Figure 8. The touch trigger probe had a ball tip dimeter of 3 mm and a 50 mm overall length. The components with dimensions of up to 1200 × 900 × 700 mm could be handled by this machine. Inspection points were recorded on the specimen to ascertain how much the fabricated specimen deviated from the design specifications. The measuring part had to be perfectly fixed on the machine table, and the machine must be clean. In addition, three measurements were taken on each sample to guarantee the accuracy of the results. Each measurement process commenced with the CMM being calibrated against a standard sphere artifact of predefined size. A rectangular plate of 127 × 12.7 × 3.2 mm and a cylinder specimen with a diameter of 12.7 mm and height of 25.4 mm were used to compute the shrinkage percentage in each material.

**Figure 9:**
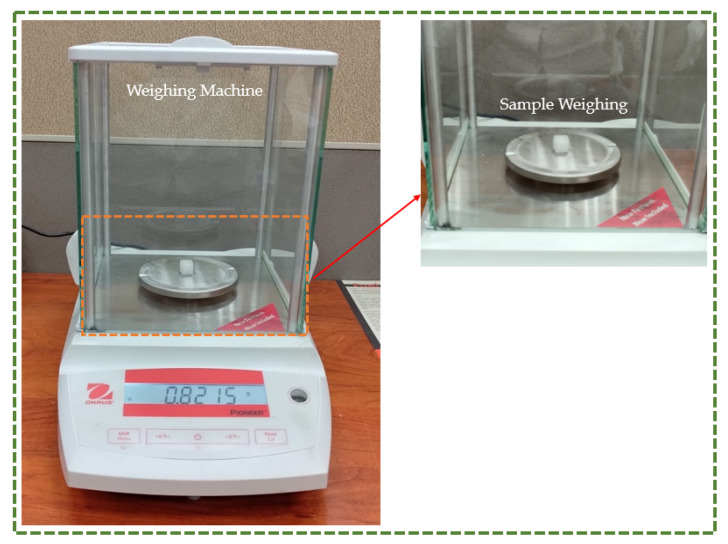
Setup to measure weights.

A cube (10 × 10 × 10 mm), a rectangle plate (20 × 10 × 2 mm), and a cylinder (5 × 20 mm) were 3D printed to determine how much water each material can absorb. The 3D printed samples were first dried in an oven and, following that, they were submerged in water at room temperature for 0.5, 2, 12, 24, 72, and 192 h [48,49]. Equation (1) was used to determine the water absorption of the printed samples, where W_i_ is the initial weight prior to water immersion and W_f_ is the final weight following water immersion. Figure 9 portrays the weight measuring setup.

(1) $$\mathrm{Water}\;\mathrm{absorption}\;(\%)=\frac{W_{i}-W_{f}}{W_{i}}\;\text{×}\;100$$

## 3. Results and Discussion

**Figure 10:**
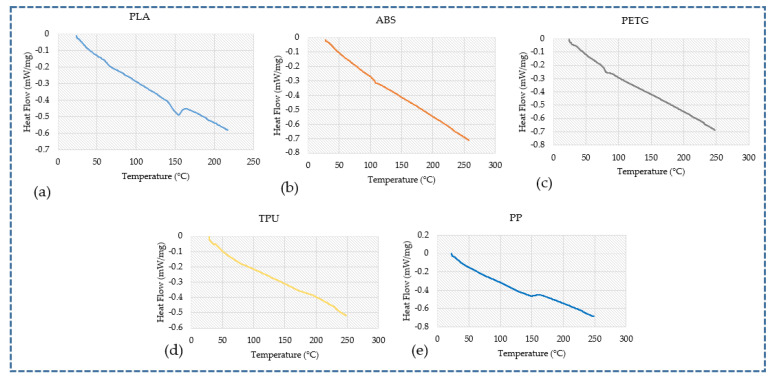
DSC curves for different polymers: (**a**) PLA, (**b**) ABS, (**c**) PETG, (**d**) TPU, (**e**) PP.

The thermal characteristics, T_g_ and T_m_, were examined by DSC investigations. The thermograms of PLA, ABS, PETG, TPU, and PP from DSC tests are displayed in Figure 10. Transitions (representing T_g_) in the thermograms were seen at 63.2 °C, 103.35 °C, and 78.7 °C for PLA, ABS, and PETG, respectively. These outcomes are in line with the supplier’s technical data sheet, which was provided by Raise 3D Technologies Inc. The T_g_ (as provided by the supplier) for PLA, ABS, and PETG were 61 °C, 101 °C, and 81 °C, respectively [50,51,52]. The glassy state of the polymers changed to a rubbery form at this temperature (T_g_) [53]. The DSC curves, seen in Figure 10, also illustrate that there was no conclusive T_g_ for TPU and PP. Furthermore, the T_m_ for PLA and PP were 153.6 °C and 147.1 °C, respectively; however, the T_m_ for ABS, PETG, and TPU were indeterminate [32,50,54,55]. The T_m_ for PLA and PP, as provided by the supplier data sheet, are 150 °C and 137 °C, respectively, and are quite closer to the experimental values [50,55].

**Figure 11:**
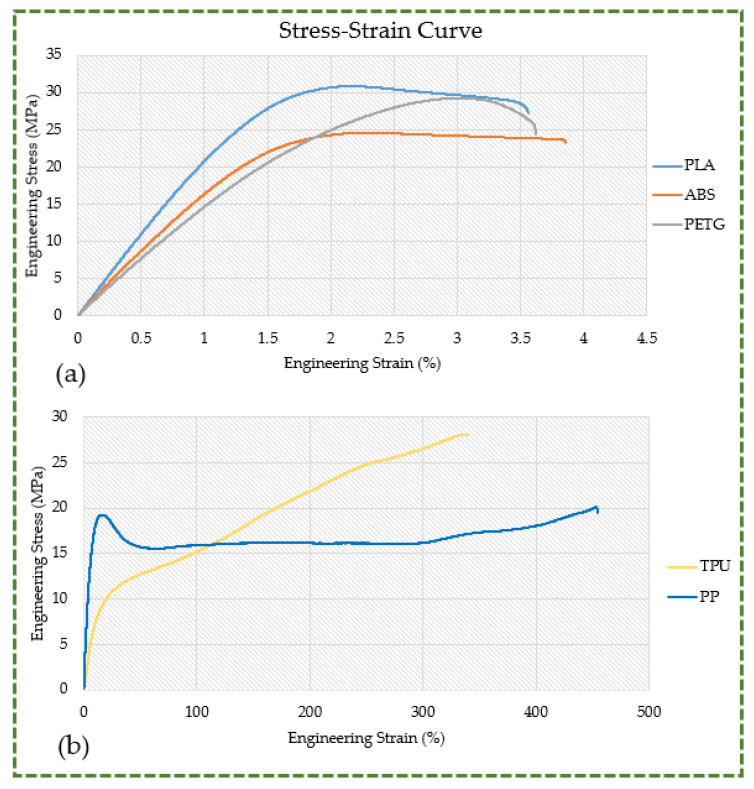
Stress–strain curves (sample 1): (**a**) PLA, ABS, and PETG; (**b**) TPU and PP.

**Figure 12:**
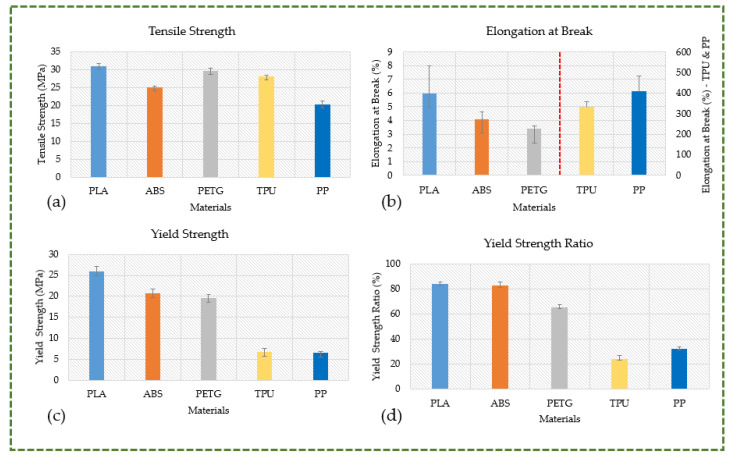
Comparative analysis: (**a**) tensile strength; (**b**) elongation at break (TPU and PP are on a separate *Y*-axis); (**c**) yield strength; (**d**) yield strength ratio.

Figure 11 shows the stress–strain curves for samples of PLA, ABS, PETG, TPU, and PP. When it comes to PLA, there occurred some plastic deformation in the vicinity of 2% strain, and it was yielded to a breaking strain of 3.56%. The ABS curve has a region of elastic and plastic deformation that exhibits stress–strain behavior which is initially linear under tensile stress like that of PLA. Additionally, the elongation at the break of ABS (3.86%) was just marginally greater than that of PLA. The resistive stress was observed to be nearly constant, with an increase in strain through the shape until the ABS sample reached its maximal stress. It is also observed in Figure 11a that ABS has almost 20% lower strength than PLA. Additionally, the analysis demonstrates that PLA and ABS are both brittle and rigid, with their modest plastic deformations indicating higher resistance to deformation and a propensity for sudden rupture. It has also been established through comparison that PLA is somewhat less brittle than ABS as well as PETG (refer to Figure 12b). Compared to PLA and ABS, PETG has the lowest elastic modulus, which is the gradient of the stress–strain curve in the elastic region. In addition, PETG is as strong as PLA, but it does not extend as much when it reaches its yielding point, instead becoming highly brittle. Given that PETG does not undergo plastic deformation, there is a very high likelihood of catastrophic failure. As illustrated in Figure 11b, TPU exhibits its usual mechanical behavior as predicted, together with a steadily rising tensile strength and elongation until the break. Its characteristics include an extremely low Young’s modulus, which makes it flexible and soft, tensile strength that is on par with PLA, and almost no tendency toward catastrophic failure. The PP specimen displayed the usual stress–strain characteristics of tough and ductile polymers. The pure elastic deformation behavior was characterized by an almost linear rise in stress at low strain. This was the result of yielding, which is demonstrated by the stress–strain curve’s departure from linearity at a strain of roughly 12%. Here, the polymer chains began to slip, and strain hardening occurred as the strain level rose, eventually resulting in the breaking of PP. In comparison to other polymers, the yielding in PP is very noticeable. However, there was no discernible yielding in the cases of PETG and TPU.

With the exception of PP, all materials have tensile strengths in excess of 24 MPa, which qualifies them for use in structural parts or components that require more strength and rigidity (see Figure 12a). With a greater tensile strength, PLA is the strongest material; PETG and ABS are the next strongest materials. As seen in Figure 12b, all three of these materials have the same combination of reduced elongation at break and higher tensile strength. The most brittle material is PETG, followed by ABS and PLA. This combination illustrates that although these materials can withstand significant tensile stresses, they do not undergo significant deformation before collapsing. When under stress beyond their limit, they may fracture suddenly and without yielding, demonstrating an insufficient capacity to absorb energy prior to fracture. Although they are strong, their unpredictability and inability to deform before failing prevent them from being used in scenarios where stronger materials are needed or where the application demands some degree of deformation before breaking. As depicted in Figure 12b, TPU exhibits a high elongation at break (332.10%) and an elevated tensile strength (28.07 MPa). Its mechanical characteristics are distinct, with greater tensile strength, high elongation at break, and a less apparent yielding point. This blend shows that TPU can stretch extensively prior to final breakdown and withstand large forces. The yielding point appears to be less prominent, indicating a smoother and steady transition from the elastic to plastic region. It finds utility in instances when ductility and strength are essential. PP exhibits the highest elongation at break (407.99%), lowest tensile strength (20.23 MPa), and most pronounced yielding point of all the materials. While it can deform extensively before rupturing, it does not have the same level of resistance to external forces as the other materials under consideration. At about 12% strain, PP starts to irreversibly deform, as evidenced by the more pronounced yielding point, which shows a definite behavioral change under tension. Likewise, as illustrated in Figure 12c, PLA has the highest yield strength (25.98 MPa), followed by ABS (20.70 MPa), PETG (19.55 MPa), TPU (6.75 MPa), and PP (6.58 MPa). This implies that PLA is able to endure higher stress levels before experiencing plastic deformation or irreversible shape change. TPU and PP, on the other hand, plastically deform even under light loads, making them inappropriate for components that need to retain their fit and shape. Given the importance of knee orthoses and other custom orthoses built to fit the unique anatomy of the wearer, the material used in their construction must have a higher yield strength. The yield strength ratio, as seen in Figure 12d, was additionally examined for various materials, much like the yield strength. It explains the maximum amount of elastic stress that a material can withstand. PLA, in comparison to materials like TPU and PP, which have lower ratios, has superior resistance to permanent deformation under load due to its higher yield strength-to-tensile strength ratio. The greater yield strength ratio of PLA indicates that its tensile strength—the highest stress it can bear before breaking—and yield strength—the stress at which it starts to deform plastically—are nearly similar. Thus, PLA is more resilient to stress before it indefinitely deforms than it is at its point of failure. Conversely, TPU and PP have lower yield strengths and relatively higher tensile strengths, which accounts for their lower yield strength ratios.

It is also essential to emphasize that injection-molded specimens can acquire a level of strength that 3D printed samples cannot match. There are a number of causes for this, the most important of which is the improper printing strategy. As reported in [56], the tensile strength of the 3D printed flat-based samples with 100% infill was 69% that of the injection-molded samples. ABS that was 3D printed had a tensile strength of 28.35 MPa, whereas ABS that was injection molded had a tensile strength of 40.82 MPa. According to the findings from another study, 3D printed ABS tend to have tensile strengths between 65% and 72% of those of injection-molded ABS [57]. Similarly, as stated in [58], the tensile strength of FDM-printed PLA was found to be 48% lower compared to those fabricated using injection molding. It was discovered that 3D printed PETG’s tensile strength ranged from 83% to 96% that of the PETG strength obtained through injection molding. Additionally, it has been demonstrated that the tensile strengths of TPU specimens produced using an optimal 3D printing method were 95% that of TPU components manufactured using an injection molding process [59]. This was further established by another investigation wherein the 3D printed PP specimens’ tensile strength ranged from 59% to 61% that of the injection-molded PP samples [60].

As injection molding is performed at a high pressure, desirable polymer chain connection is encouraged, resulting in specimens with greater strength than those produced by 3D printing [61]. Additionally, when the mold is filled, the temperature within the barrel rises, causing a symmetric flow that raises the samples’ modulus [62]. Conversely, in the layer-by-layer FDM process, the filaments typically fail to adhere and connect to one another properly. This causes the filament strands to generate spaces and open structures with large gaps between them, which reduces the strength of the component [63,64,65]. Moreover, the components fabricated by FDM have anisotropic behavior that heavily relies on the raster patterning and air gap [57,66]. Undoubtedly, one of the advantages of 3D printing is its ability to efficiently create intricate shapes, including those involving human anatomy. When it comes to creating unique shapes and geometries, 3D printing is the ideal option. Because of this, choosing the right print parameters for a given material is essential when using 3D printing.

Additionally, the recorded tensile strengths were compared with the tensile strengths obtained from each material’s technical data sheet [50,51,52,55,67]. According to their technical data sheets, PLA, ABS, PETG, TPU, and PP have tensile strengths of 46.6 MPa, 33 MPa, 31.9 MPa, 29.3 MPa, and 35.1 MPa, respectively. All the polymers had tensile strengths that are marginally less than those found on the technical data sheet. The specimens in this work were printed at 45°/135° raster orientation, but the reference specimens were stated to be printed at 0°, which may account for the slightly lower strengths found in this work. Compared to other polymers whose values fall within a comparable range as specified in the technical data sheet, the tensile strength of PP was significantly lower. It can be explained by the fact that, in contrast to other polymers, PP’s strength is strongly influenced by the raster orientation. For example, when PP is printed at 90°, its tensile strength is approximately 50% that of PP with a 0° raster orientation [55]. This finding highlights that raster orientation—45°/135° in this instance—has a greater impact on PP’s tensile strength than it does on other polymers.

The results are further validated by citing findings from the literature where the various polymers were compared. In their comparison of the various polymers, Petersmann et al. [68] found that PLA had the highest tensile strength, followed by PETG and PP. PLA also showed greater strength in comparison to TPU [69]. As stated in [70], when the components were built at a 45° raster angle, PLA resulted in the maximum tensile strength as compared to ABS and PETG. Additionally, among a number of polymers (PLA, ABS, PETG, TPU, etc.), PLA had the maximum tensile strength, according to Sedat et al. [71]. Further, test specimens made of PLA outperformed ABS in terms of rigidity and tensile strength, as demonstrated by Rodríguez-Panes et al. [72] and Priya et al. [73]. While comparing PLA, ABS, and PETG, Pernica et al. [74] found the highest strength in PLA and the lowest tensile strength in PETG.

**Figure 13:**
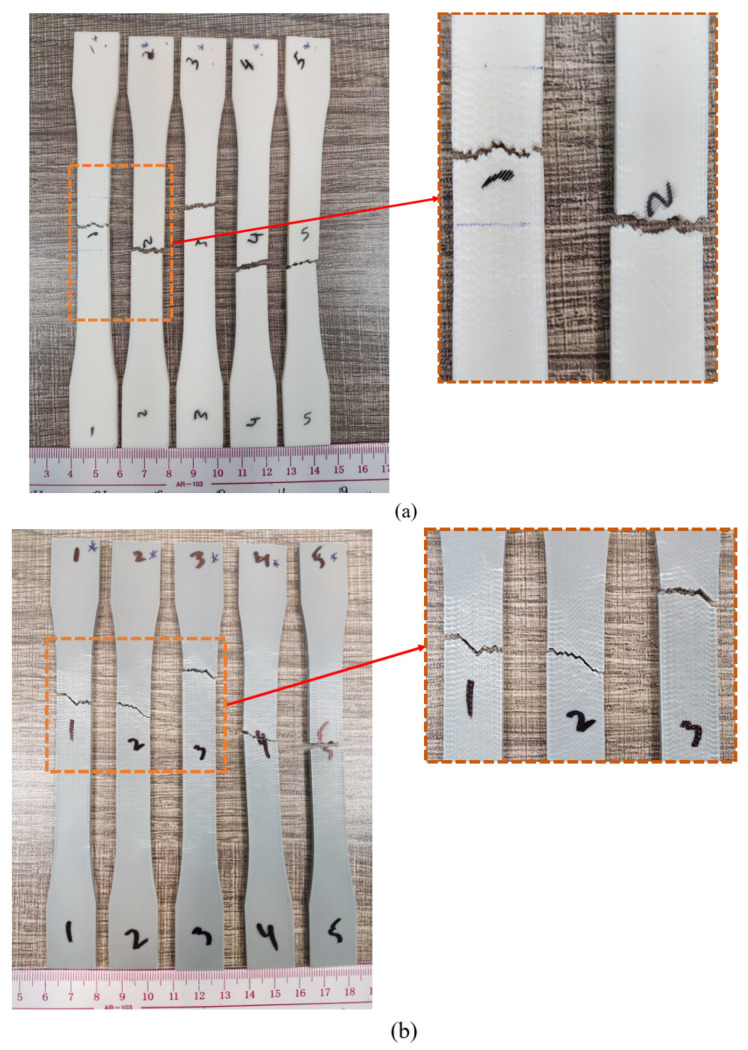

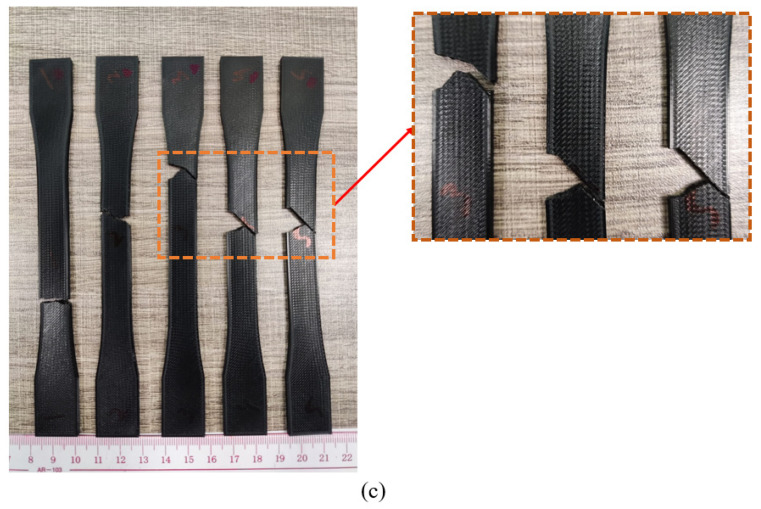
Fracture incidents in different materials. (**a**) Failure of PLA due to in-layer fracture and along the center. (**b**) ABS breakage due to a combination of in-layer and inter-layer fracture mechanisms, mostly in the center with a few exceptions. (**c**) PETG fails with inconsistent fracture locations and an inter-layer fracture mechanism.

Based on the findings presented in Figure 13a, it appears that all PLA samples failed in the vicinity of their centroid during tensile testing, indicating a centralized or median fracture mechanism. This implies that the stress was distributed uniformly across PLA’s cross-sectional area. When stress surpassed its threshold in PLA, failure took place, resulting in an instantaneous fracture. This also suggests that PLA had experienced uniformly distributed tensile tension throughout the specimen and that there were no apparent localized flaws or areas of concentrated stress. Similar to PLA specimens, all ABS samples (shown in Figure 13b) had fractures primarily in the middle region, with a few exceptions where the fracture was somewhat over the center region, close to the top grip. This implies that there may have been a flaw in ABS that caused the fracture to start at this specific location. The fracture spot kept varying in PETG samples, as evident in Figure 13c. This underlines the possibility of stress concentration and the fact that PETG’s characteristics are not consistent throughout its structure. Furthermore, there could be voids or tiny cracks that serve as the starting points for fractures at different points.

**Figure 14:**
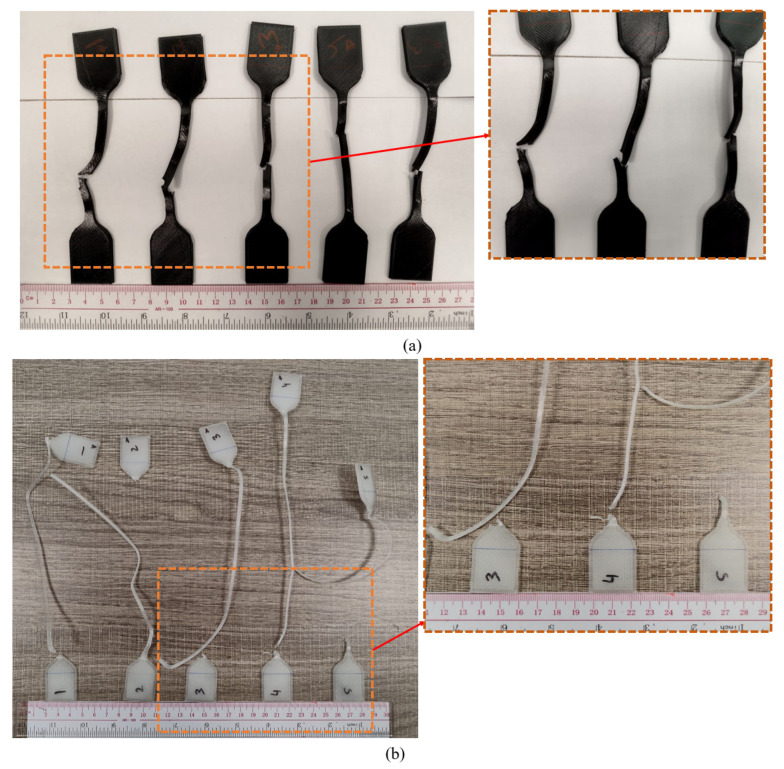
Fracture occurrence in highly flexible materials. (**a**) TPU fractures along the center and via an inter-layer fracture process. (**b**) PP fracturing near the bottom and with inter-layer fracture.

As discussed above, TPU is a highly elastic and flexible material with a complicated fracture process. The fracture in TPU occurred close to the central location, as depicted in Figure 14a. In other words, this points out that TPU responded to the imposed load consistently and did not exhibit any notable localized flaws or vulnerabilities. Referring to Figure 14b, practically all of the samples show that PP consistently failed close to the bottom grip. It is quite probable that there were some cracks or anomalies close to the bottom grip region which contributed to the stress concentration and turned the area into a fracture site. From the fracture results in Figure 14, it can also be inferred that TPU and PP experienced considerable elongation prior to failure. Additionally, necking involving localized deformation and a reduction in cross-sectional area was seen in both materials. Their high plastic deformation also indicates that they have a high energy absorption capacity during deformation, which aids in their resistance to fracture and enables them to flex significantly before failing, preventing any severe rupturing [53,75,76].

**Figure 15:**
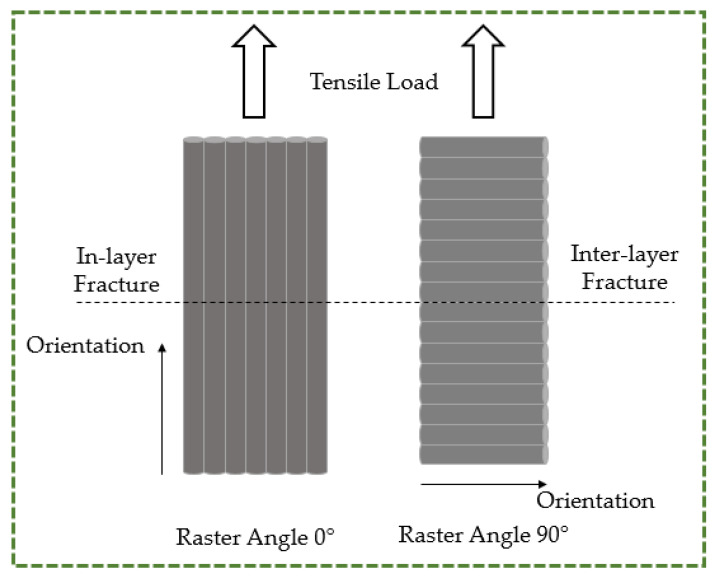
Types of tensile failure modes.

Prior to delving into the specifics of the fracture phenomenon, it is imperative to comprehend the various potential failure mechanisms. According to previous examinations of tensile failure, it is observed that inter-layer and in-layer fracture modes occur in the specimens following tensile tests [77]. Therefore, to properly analyze the nature of the tensile experiment results, the two failure modes—the in-layer and the inter-layer failure modes—that are depicted in Figure 15 had to be characterized [78]. When two adjacent material layers fracture at their interface but the material layers hold together after the failure, and the angle between the failure surface and the material layers is zero, it is known as an inter-layer failure mode. When material layers fracture and there is an angle that is not equal to zero between the failure surface and the fractured material layers, an in-layer failure mode takes place.

The fracture profiles of PLA, ABS, and PETG appear to zigzag, as seen in Figure 13. The existence of zigzag patterns in these materials indicates brittle fractures and minimal ductility. Sharp, angled surfaces are seen in the fracture zone, and the fracture seem to be growing in a swift erratic manner. The direction of the applied force appears to be perpendicular to the fracture propagation in PLA, and the angle between the fracture surface and the raster directions is non-zero, reflecting a dominant in-layer failure mechanism. Therefore, the failure mechanism depends primarily on the strength of each individual fuse or fibril inside the raster layer rather than the bonding between the adjoining raster layers. Because the applied tensile load was 45° from the direction of the raster layers, the tensile load was divided into two component forces, namely parallel and perpendicular to the direction of the raster layer. The failure mechanism of the specimen is in accordance with the 90° raster angle (inter-layer fracture) when the specimen is dominated by the perpendicular load; conversely, when a parallel load is applied, the failure mechanism is consistent with the 0° raster angle (in-layer fracture). Referring to Figure 13b for ABS, the fracture propagation is a result of both in-layer and inter-layer fracture phenomena. A range of 0 to 45° is observed in the angle formed by the fracture surface and the raster direction. The failure of individual material strands or fibers as well as the delamination of layers from bonded sites have both contributed to the failure of ABS. As is evident from Figure 13c, the tensile testing of PETG produced an angle of 0° between the raster direction and the fracture surface. This suggests that there was a predominant inter-layer fracture brought on by bound layers’ delamination. It is apparent when analyzing the fractures of PP and TPU that inter-layer fracture was the primary reason for these materials’ failure. The reason for this is that there is typically a 0° angle between the fracture surface and the raster direction (Figure 14a,b). It is rare for individual rasters or fibrils to fail before debonding or delamination because these materials are very tough and ductile.

**Figure 16:**
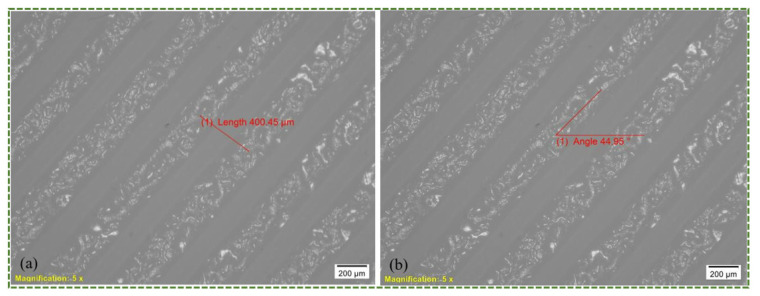
Depiction of applied (**a**) raster or extrusion width; (**b**) raster angle (top layer 45°).

**Figure 17:**
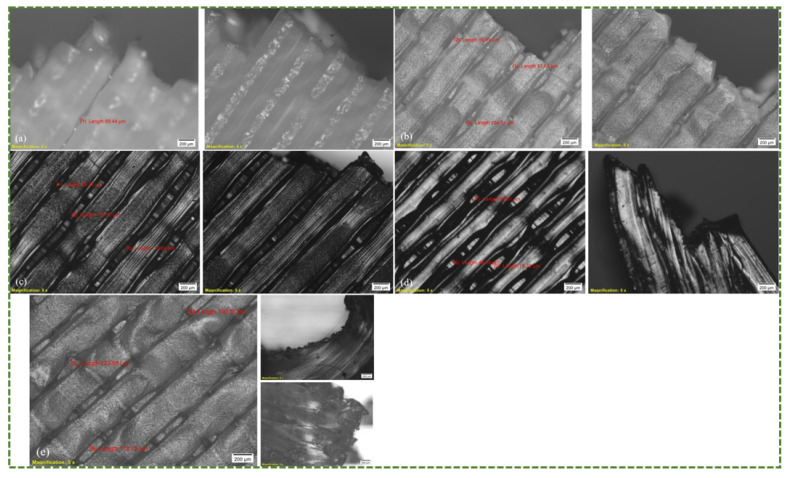
Microscopic fracture analysis (**a**) PLA; (**b**) ABS; (**c**) PETG; (**d**) TPU; (**e**) PP (scale bar for all figures is 200 μm).

The microscopic view of the as-built 3D printed PLA specimen is displayed in Figure 16. The reason for displaying this is to provide an image of the raster width, which was 0.4 mm in Figure 16a, and raster direction, which in this study was 45°/135°. As seen in Figure 16b, the angle on the top raster layer is 45°. Figure 17 shows the morphologies of the fracture surface of PLA, ABS, PETG, TPU, and PP.

**Figure 18:**
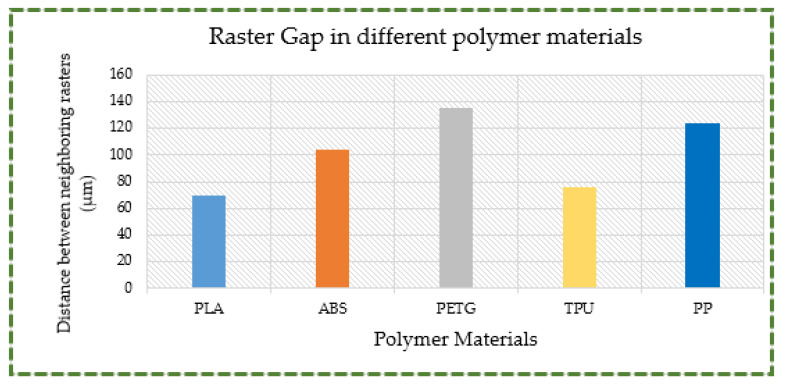
Distance between two neighboring rasters for different polymers.

A glance at the PLA microscopic pictures also reveals a prominent in-layer fracture brought on by the fracturing of individual rasters or fibrils instead of delamination. As seen in Figure 17a, the maximum distance between two neighboring rasters is roughly 69 μm in the vicinity of the fracture site. Furthermore, it is evident that PLA has a brittle behavior with a zigzag pattern that includes sloped and pointed surfaces. In contrast, Figure 17b shows that, in the ABS material, the separation between two successive rasters is rather large in close proximity of the fracture. There is a maximum gap of about 100 μm, as depicted in Figure 18. Consequently, as has previously been discussed, the combination of in-layer and inter-layer factures caused the ABS material to fail in the tensile test. This includes both the separation of rasters from one another and the breaking of individual rasters. Furthermore, it can be seen from Figure 17c that inter-layer fracture was the predominant tensile failure mode in the PETG specimens. The gap between the two rasters is more than 135 μm in near proximity to the fracture site. Thus, the main cause of the PETG failure was the separation of two successive rasters, which was followed by the breaking of individual rasters. It is apparent (see Figure 17d,e) that inter-layer fracture was the main cause of failure for TPU and PP due to their ductile and pliable characters. The materials continued to stretch until the final fracture occurred because of their increased plastic deformation as well as ductile and elastic nature when the load increased. As illustrated in Figure 18, the gaps between two successive rasters for TPU and PP are more than 76 and 123 μm, respectively. It can therefore be deduced that the nature of material fracture is influenced by a number of variables, including the raster distance and the flexibility or rigidity of the material. Greater raster distance indicates a lack of strong bonding between the rasters and increases the likelihood of inter-layer facture. Likewise, flexible materials are more likely to have inter-layer fracture because they will continue to expand as a result of their flexibility, ultimately failing mostly through raster delamination or separation.

Optimizing printing conditions, post-processing, etc., are some of the ways to mitigate inter-layer fracture. Enhancing inter-layer adhesion for a particular polymer can be achieved by determining the ideal combination of printing parameters, such as temperature, speed, layer height, raster direction, etc. Additionally, post-printing heat treatments can minimize residual stresses, increase inter-layer bonding, and enhance crystallinity—all of which lessen the chances of part failure. Since fabrication defects serve as the activation points for in-layer fractures or stress concentration, it is crucial to ensure that they are insignificant to reduce in-layer fracture. This can be accomplished by choosing the printing settings judiciously. Furthermore, it is crucial to design components with smooth transitions, fillets, etc., rather than aggressive edges or corners.

Moreover, by comparing the failure surfaces of injection-molded and 3D printed specimens, the following conclusion can be made for a deeper understanding. Because injection-molded samples are homogeneous, exhibit isotropic behavior, and lack layer-by-layer construction, they often have a smoother fracture failure cross-section than 3D printed ones [79]. There are no noticeable surface imperfections or layers, and an injection-molded part’s failure surface is usually uniform and continuous. Additionally, Wijdan [80] has shown that, in contrast to 3D printed ABS, which broke in a brittle way, injection-molded ABS experienced necking prior to tensile failure.

Polymers used in knee orthoses can degrade over time due to water absorption [81,82,83]. This is because water absorption results in a reduction in a polymer’s mechanical properties, including strength, rigidity, durability, etc. As a consequence, the capacity of a knee orthosis to adequately support, stabilize, and shield a knee joint is impacted. Water absorption can also create an environment that is conducive to the growth of microorganisms, which could result in problems such as contamination by bacteria or fungi [84]. This renders the user susceptible to infections or skin irritations and raises hygiene issues. Furthermore, the fitting of the orthosis is affected because the polymer alters its shape over time due to water absorption [85]. This causes discomfort for the user and decreases the orthosis’s ability to effectively align and hold up the knee joint. Therefore, it is critical to comprehend how various 3D printed polymer materials behave in terms of their propensity to absorb water.

**Figure 19:**
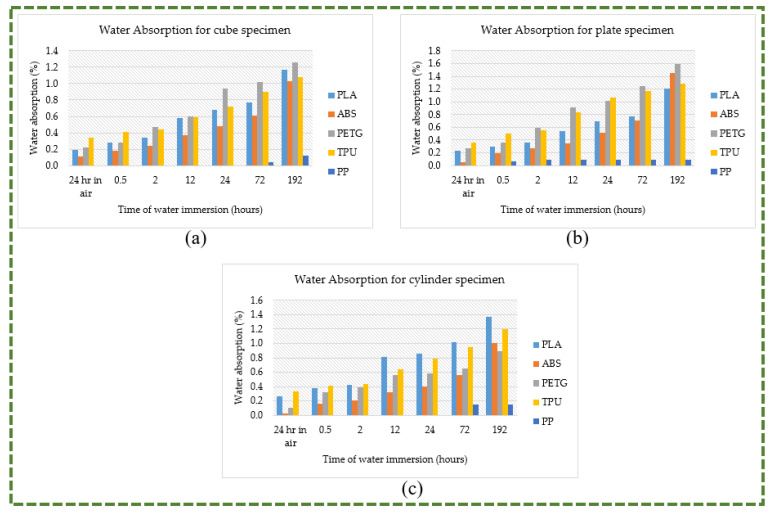
Propensity of water absorption for different specimens (**a**) Cube; (**b**) Plate; (**c**) Cylinder.

The water absorption percentages for various polymers using different geometries, following a 24 h period of exposure to air and 0.5, 2, 12, 24, 72, and 192 h of submersion in water, are shown in Figure 19a–c. Of all the polymer materials under consideration, PP had the lowest proportion of water absorption. When PP was left outside for a full day (in open air), it did not absorb any water. Likewise, it was found that cube and cylinder specimens only absorbed some water after being submerged in water for 72 h uninterrupted. There was a very small increase in the percentage of water absorption by PP even after 192 h of immersion in water. In terms of water absorption upon exposure to air, ABS comes in second on the list. It only absorbed a maximum of 0.11% of the water. PLA, PETG, and TPU absorbed 0.26%, 0.27%, and 0.36% of the water, respectively. Additionally, it is noted that, with the exception of PP, all the polymers exhibited a steady increase in water absorption percentage with increasing immersion time. Additionally, it is shown that PETG had the greatest percentage of water absorption (1.59%) after 192 h of soaking in water, followed by ABS (1.44%), PLA (1.37%), and TPU (1.29%). All of the polymer materials were discovered to have significant hydrophobicity even after being submerged in water for 192 h nonstop. They have good water resistance, and the presence of moisture in the environment barely affects their performance. Thus, these polymer materials’ inability to absorb water allows for them to be utilized successfully as a material for knee orthoses.

The repeated heating and cooling cycles that occur throughout the 3D printing process result in the shrinkage of 3D printing polymers [86,87]. As a result, a finished 3D printed component has inaccurate dimensions. Therefore, it is essential to investigate the percentage of polymer shrinkage that occurs during the 3D printing process before using polymers in actual applications. If the dimensions of a 3D printed knee orthosis significantly deviate from the design specifications, the orthosis will not fit the wearer correctly and will cause discomfort.

Thus, it is crucial to comprehend the shrinkage performance of the various polymers and account for an appropriate tolerance when designing knee orthoses. Additionally, it is essential to choose a material with the least amount of shrinkage possible to reduce revisions and user distress.

**Figure 20:**
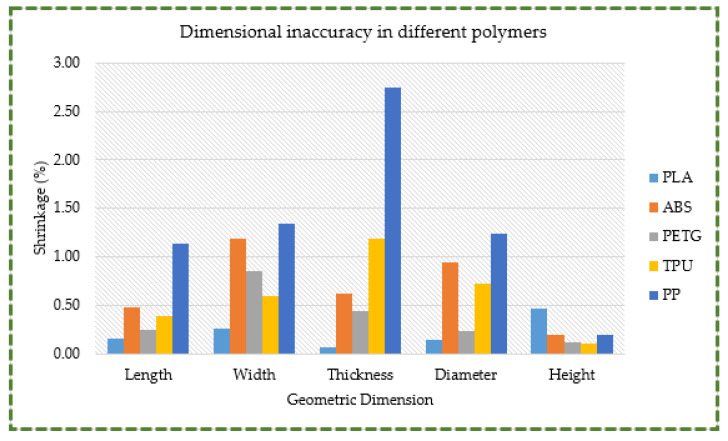
Dimensional inaccuracy observed in different materials.

PLA was found to have the least amount of shrinkage among the polymer materials under consideration, while PP exhibited a considerable amount of shrinkage (refer Figure 20). PLA had the highest shrinkage percentage (0.46%), followed by PETG (0.85%), ABS (1.18%), TPU (1.19%), and PP (with the highest shrinkage of 2.75%). The low shrinkage percentage in PLA can be ascribed to several variables. For example, its nozzle temperature is 205 °C, whereas PP’s nozzle temperature is 220 °C. Since the difference in temperature between melting and cooling cycles is smaller, there is a reduced shrinkage in PLA through the solidification process. Furthermore, it has been observed that PLA has a lower coefficient of thermal expansion (61 μm/(m·°C)) than PP (101 μm/(m·°C)) [88]. For a given change in temperature, a material with a low coefficient of thermal expansion will expand (or contract) less than a material with a greater coefficient of thermal expansion. Thus, when PLA cools from its molten state, it contracts less, leading to lower shrinkage. 

Based on the summary in Table 2, it can be inferred that each material has advantages and disadvantages. This implies that all the desired functions cannot be obtained from a single material. For instance, while ABS has good mechanical qualities, it also absorbs water, making it unsuitable for use in orthoses. Comparably, PP absorbs water the least but has a poor yield strength and is challenging to print. As a result, it is critical to compromise on one or two properties and consider a property that can provide the required functionality.

It can be observed that PLA outperforms all the other materials taken into consideration in the majority of the attributes, such as yield strength, tensile strength, shrinkage percentage, cost, and ease of printing. Even though its percentage of water absorption is slightly higher, it is still lower than that of ABS and PETG. TPU and PP were not considered because of their very low yield strength. With low yield strengths, their ability to regain their original shape also reduces, which in turn affects the comfort fitting of an orthosis. In addition, their shrinkage percentages are quite high. Similarly, ABS and PETG have, more or less, the same properties in comparison to PLA, but the major differences are the biodegradability and biocompatibility of PLA. Furthermore, the shrinkage percentage in PLA is lower than those of ABS and PETG; therefore, there is a lower possibility of dimensional error in the manufactured specimen. This is crucial for ensuring that an orthosis fits properly. It is certainly feasible to further demonstrate here that the findings of this study are in accordance with those of earlier studies in the literature, as Table 3 illustrates.

**Figure 21:**
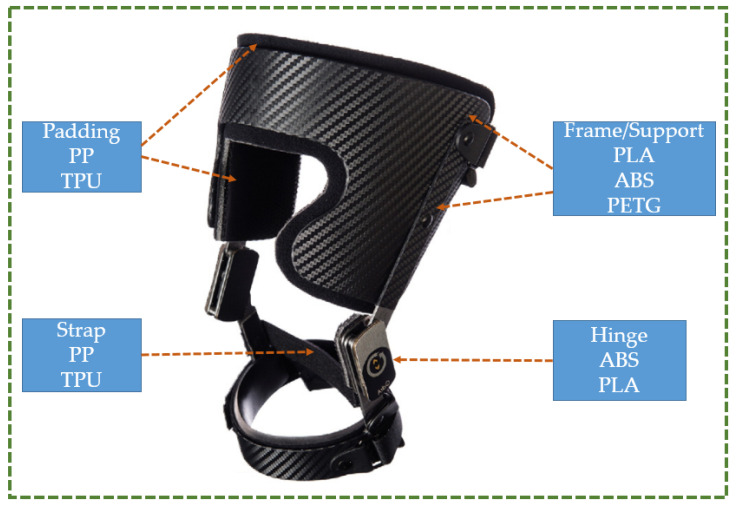
Application of different polymer materials in knee orthosis [96].

There is also the possibility that a combination of materials can be used in designing knee orthoses. A knee orthosis is composed of multiple components including structural elements that form the frame of the orthosis, moving parts such as hinges, padding to protect the user from the rigid elements, and straps or fastening elements. The different components are shown in Figure 21, and this illustration of a knee orthosis is derived from Laroche et al.’s study [96]. The aforementioned study suggests that PLA is the best material to use to fabricate structural components because of its enhanced rigidity and stiffness, low shrinkage factor, and convenience in printing. The frame is made to fit the unique shape of a human’s leg, and thus while the outside shape can be intricate, it can be easier to print with PLA. Additionally, either PLA or ABS, which have a higher toughness as compared to PETG, can be utilized for fabricating hinges and other moving parts. If PLA is not available, ABS and PETG can also be utilized for the frame or structural parts because they can still offer an adequate level of strength. While both PP and TPU can be utilized as padding, PP should be chosen. The reason for this is that cushioning material comes into direct contact with the skin, and PP will not irritate it because it is biocompatible. Furthermore, because of its hydrophobic nature, it will not be impacted by skin sweating. It can also shield PLA, ABS, or PETG from moisture evaporating from the skin because it is placed between the skin and the structural material. Additionally, TPU and PP offer good shock-absorbing qualities, flexibility, and anatomical adjustment to the knee. Moreover, because of their flexibility, PP and TPU can be utilized for straps.

## 4. Conclusions

Material selection is a crucial design consideration for any component. Negative effects and unwanted functionality are more common than beneficial consequences when an improper material selection is made. It is essential to conduct a complete analysis of the materials before deciding on one, for the purpose of selecting the right material. With this objective in mind, work is being conducted to adopt an efficient process that can be useful to choosing the appropriate material for 3D printing a knee orthosis. Various materials’ characteristics have been examined and evaluated. Thermal examination, mechanical tests comprising tensile testing, microscopic examinations, a study of water absorption, an assessment of shrinkage factors, and dimension accuracy analysis have all been carried out. It is recognized that every material has advantages and disadvantages, and that no single material can offer every benefit. As a result, the right material must be chosen in accordance with the intended application and the resources available.

The study conducted in this paper indicates that PLA has the highest yield strength as well as tensile strength, and lowest shrinkage percentage. Out of all the materials taken into consideration, PLA, ABS, and PETG are the least expensive and easiest to 3D print. Similarly, PP is highly hydrophobic, exhibits the highest shrinkage percentage, and has the largest elongation at break. Because of their increased flexibility, PP and TPU have proven to be particularly challenging to 3D print. When the attributes of several materials are compared, it is discovered that PLA is the best material for the device’s frame, while ABS or PLA can be utilized for a knee orthosis’s hinges and moving components. TPU and preferably PP can be used as padding material as well as straps.

This work focused on the selection of appropriate material for the 3D printing of knee orthoses. The future task will be to design a knee orthosis using a user’s anatomy information, fabricate the various components using the recommended materials, and mount it on the patient’s knee to evaluate its performance. Additional mechanical tests, such as compression and flexural testing, will be a part of the upcoming work.

## Figures and Tables

**Table 1:** Variables set for fabricating different materials.

Material	Parameters					
	Heated Bed Temperature (°C)	Nozzle Temperature (°C)	Speed (mm/s)	Layer Height (mm)	Infill (%)	Extrusion Width (mm)
PLA	55	205	70	0.1	100	0.4
ABS	100	250				
PETG	60	245				
TPU	60	225	50			
PP	70	220	60			

**Table 2:** Mechanical and Physical properties measured for different polymers.

Material	Yield Strength	Tensile Strength	Elongation at Break	Water Absorption (%)	Shrinkage Percentage (%)	Ease of Printing	Cost (USD)/Kg
PLA	25.98	30.89	5.96	1.37	0.46	Easy	34.99
ABS	20.70	24.99	4.07	1.44	0.85	Easy	34.99
PETG	19.55	29.72	3.38	1.59	1.18	Easy	34.99
TPU	6.75	28.07	332.10	1.29	1.19	Difficult	79.99
PP	6.58	20.23	407.99	0.15	2.75	Difficult	59.99

**Table 3:** Summary of previous research findings which corroborate the conclusions of the current study.

Study	Objective	Findings
Petersmann et al. [68]	Six distinct 3D printable polymers—PEEK, PLA, PMMA, PETG, PVDF, and PP—that could be useful in medical applications were examined for their tensile properties.	Results showed that PLA had the maximum tensile strength when compared to PETG and PP.
Rahmatabadi et al. [69]	Three PLA-TPU composites were compared for their mechanical properties.	Strength and printability of the PLA-TPU combination diminished as the proportion of TPU increased.
Kopar and Yildiz [70]	Mechanical characteristics of materials made of PLA, PETG, and ABS were investigated at different raster angles.	Maximum tensile strength was observed in PLA components manufactured at a 45° raster angle.
ÇELİK et al. [71]	Evaluated the strength of PLA, ABS, PETG, TPU, and ASA filaments.	PLA had the highest tensile strength of all the materials taken into consideration.
Rodríguez-Panes [72]	Explored the mechanical properties of ABS and PLA parts.	PLA test specimens performed better than ABS in terms of stiffness and tensile strength.
Pernica [74]	Tensile characteristics of PLA, PETG, and ABS manufactured at ±45° were studied.	PLA showed the greatest strength while PETG had the lowest tensile strength.
Ngaowthong et al. [89]	Investigated the recycled PLA’s and PP’s propensity to absorb water.	Water absorption was higher in PLA than in PP.
Butylina et al. [90]	Investigated the mechanical characteristics and water absorption of composites composed of softwood sawdust and polymers like PLA and PP.	Compared to PP composites, PLA composites absorbed more water.
Spoerk et al. [91]	Evaluated the techniques to reduce shrinkage in PP parts produced via 3D printing.	Established that the primary drawback of PP in 3D printing was its extreme proneness to shrink. Further discovered that PLA was 3D printed easily due to its significantly lower shrinkage tendency.
Milovanović et al. [92]	Analyzed the effects of printing factors on PLA’s mechanical characteristics.	Stated that PLA shrunk less than ABS.
Irshad Ullah et al. [93]	Studied the shrinkage and dimensional accuracy in ABS, PLA, and PETG.	PLA showed the lowest overall average shrinking percentage.
Kumar et al. [94]	Explored the material deposition of ethylene vinyl acetate, a flexible material.	Revealed that it was more difficult to 3D print flexible materials than rigid ones.
Zhou et al. [95]	Six materials—ABS, PLA, TPU, PETG, polyvinyl alcohol (PVA), and polycarbonate (PC)—were investigated in 3D printed trimalleolar fracture models to figure out their attributes.	Authors noted that tha PLA and 1:1 scale replica were favored by the patients.

## Data Availability

The data presented in this study are available in the article.
